# Impact of drainage catheter material, size, and anti-dislodgement mechanism on percutaneous nephrostomy exchange intervals: a systematic review protocol

**DOI:** 10.12688/f1000research.135431.1

**Published:** 2023-10-30

**Authors:** Deepak Iyer, Menelaos Konstantinidis, Hanzhou Li, Zachary Bercu, John Moon

**Affiliations:** 1 Department of Radiology and Imaging Sciences, Division of Interventional Radiology and Image-Guided Medicine, Emory University, Atlanta, Georgia, USA; 2 Division of Biostatistics, Dalla Lana School of Public Health, University of Toronto, Toronto, Ontario, Canada

**Keywords:** Drainage catheter, exchange interval, anti-dislodgement, interventional radiology

## Abstract

**Background:** Percutaneous nephrostomy (PCN) is a commonly performed procedure by interventional radiology and urology to treat urinary obstruction. In this procedure, a catheter is percutaneously placed into the renal pelvis for urinary diversion or hemorrhagic cystitis. Material type, catheter size, and catheter shape (anti-dislodgement feature) ultimately contribute to the inherent traits of longevity in drainage catheter device. Reviewing the relative strengths or weaknesses of products in the existing clinical market may help clinicians critically appraise the devices they use with evidence-based findings from this review. Furthermore, a deeper understanding of the relative strengths and weaknesses of existing devices may help inform the next generation of drainage catheter devices to prolong the interval between exchanges without detriment to patient safety.

**Methods:** The following electronic databases will be queried: PubMed, Web of Science, Cochrane from their inception to January 2023 to identify randomized controlled trials (RCTs) and cohort studies to investigate the differences that our interventions of catheter material, size, and dislodgement mechanism will have on the exchange interval (standard of care 90 days *vs.* 60 days *vs.* 45 days *vs.* 30 days). The primary outcomes will be the drainage catheter exchange frequency.

**Ethics and dissemination:** We aim to share our findings through high-impact peer reviewed journals. As drainage catheters and minimally invasive interventional radiology procedures become more popular, it is important for healthcare providers taking case of these populations to understand which variables might optimize patient care and minimize emergent exchanges. Data will be made available to readers.

**Registration:** PROSPERO ( CRD42023432788, 16 June 2023).

## Introduction

### Description of the condition

Percutaneous nephrostomy (PCN) is a commonly performed procedure by interventional radiology and urology to treat urinary obstruction. In this procedure, a catheter is percutaneously placed into the renal pelvis for urinary diversion. Obstruction is the most common indication, accounting for 85-90% of all PCN placements. ^1^ Obstruction can lead to infection, and in turn can lead to sepsis, which increases patient morbidity and mortality. ^2^ PCNs are also required for access to nephrolithotomy. Urinary diversion in the setting of urinary leak, fistula, or hemorrhagic cystitis is another common indication of PCN tubes as a modality to reroute urine from areas of inflammation. ^2^^–^^4^

Like any other procedure, PCNs are associated with major and minor complications. The Society of Interventional Radiology’s published major complication rate is around 2-10% and includes sepsis, hemorrhage, pneumothorax, puncture of adjacent organs, urine leak, and mortality. ^5^^–^^7^ The technical success rate of PCN placement is typically at least 95% in dilated calyces without stones, making it a useful procedure for the patients with the previously mentioned indications. ^8^ Prolonged use of indwelling drainage catheters has also been associated with poor outcomes, such as sepsis and encrustation, resulting in the exchanging of these catheters. ^9^ There is no consensus in the literature regarding the optimal interval at which these drainage catheters should be exchanged, however, leading many institutions to opt for a 90-day exchange interval for percutaneous nephrostomies. While this interval is sufficient for majority of patients, previous studies have shown that there is an increasing incidence of Emergency Department visits for acute PCN exchanges, resulting in increased economic burdens on these patients and the health system. ^10^

### Description of the intervention

The size and material of the catheter used in the procedure can have an impact on success and complications rates.

While small sized catheters might be more beneficial for patient comfort, some drainage catheter literature suggests that larger bore catheters may be associated with lower rates of obstruction due to thrombosis or encrustation and malpositioning. ^11^^–^^13^ Prior research in cystostomy tubes suggests that catheter flow velocity increases significantly with increasing catheter lumen size per Poiseuille’s Law. ^14^

The type and material of catheter used in percutaneous nephrostomy can also impact the success and complications of the procedure. Two of the more commonly used tubes in PCN procedures include Malecot tubes (made of silicone) and pigtail catheters (typically made of polyurethane), each of which have different complication rates. ^15^ For example, silicone catheters have been shown to have higher rates of obstruction and dislodgement as compared to that of polyurethane catheters.

Another potential complication of percutaneous nephrostomies that might prompt an earlier than expected exchange frequency is the accidental dislodgement of the catheter, which can lead to further sequelae of obstructive uropathy. A number of anti-dislodgment mechanisms exist including pigtail “Cope” catheters, balloon retention features, and Malecot re-entry tubes. ^16^

There have been no studies to our knowledge that explore these variables in the context of PCN exchange procedure intervals.

### Why it is important to do this review

Material type, catheter size, and catheter shape (anti-dislodgement feature) ultimately contribute to the inherent traits of longevity in drainage catheter device. Reviewing the relative strengths or weaknesses of products in the existing clinical market may help clinicians critically appraise the devices they use with evidence based findings from this review. Furthermore, a deeper understanding of the relative strengths and weaknesses of existing devices may help inform the next generation of drainage catheter devices to prolong the interval between exchanges without detriment to patient safety.

## Methods

This protocol follows the Preferred Reporting Items for Systematic Review and Meta-analysis Protocols (PRISMA-P). ^17^^,^^30^ Should any modifications to the protocol be performed, they will be updated in PROPSERO ( CRD42023432788, 16 June 2023). The methods for this protocol will be modelled from previously published protocols. ^18^

### Selection criteria

**Types of studies**

We will be examining randomized controlled trials (RCTs) and cohort studies, case-control trials, case series, and observational studies comparing any two interventions against one another or against a placebo. Qualitative studies will be excluded. Only studies written in English will be included; however, we will not be excluding based on date of publication or setting.

**Types of participants**

Adults (aged 18 years or older), of any sex and ethnicity, who have received a percutaneous nephrostomy as determined by the individual study inclusion criteria.

**Types of interventions**

The major interventions that will be explored through this systematic review include catheter sizes, catheter material, and anti-dislodgement mechanisms in the context of percutaneous nephrostomy exchange frequency. As discussed previously, these three variables all contribute to complications with drainage procedures that result in earlier than expected exchanges. We will be exploring studies that discuss the complication rates associated with varying catheter sizes based on French size. We will also be examining various catheter material types, such as silicone and polyurethane, as it relates to PCN complications and increased exchange frequencies. Finally, we will be examining the role that anti-dislodgement mechanisms play in the context of drainage catheter complications and need for emergent exchanges.

**Types of outcome measures**

*Primary outcomes*

• Exchange frequency will be the primary outcome of interest in our systematic review. Currently, the optimal exchange frequency is not well established in the literature and many institutions opt for performing exchanges in a 90 day interval. Through this systematic review, we will be investigating the differences that our interventions of catheter material, size, and dislodgement mechanism will have on the exchange interval (standard of care 90 days *vs.* 60 days *vs.* 45 days *vs.* 30 days).

*Secondary outcomes*

• Morbidity
• Mortality
• Rate of sepsis
• Hospital admission and readmission rates

### Search strategy

Our search strategy aims to identify all published literature for possible inclusion in this review. We will adapt the search strategy we developed for PubMed to search the following electronic databases.

The Web of Science (WoS) and Cochrane databases will be subsequently searched.

We will restrict publications to only those published in the English language.

Our institution’s Information Specialist will search MEDLINE (PubMed; 1966 to date of search), Cochrane database, Embase (Elsevier; 1947 to date of search), Scopus (Elsevier; 1788 to date of search). Grey literature sources will also be included such as ProQuest Dissertations & Theses Global, Journal of Vascular and Interventional Radiology Supplements, and Cardiovascular and Interventional Radiology Journal Supplements.

**MEDLINE search strategy**

(((((“nephrostomy, percutaneous”[MeSH Terms] OR “nephrostomy catheter*”[Text Word] OR “percutaneous nephrostom*”[Text Word] OR “nephrostomy tub*”[Text Word] OR ((“Kidney”[MeSH Terms] OR “kidney*”[Text Word] OR “nephrotom*”[Text Word]) AND (“Catheters”[MeSH Terms:noexp] OR “catheter*”[Text Word] OR “catheters, indwelling”[MeSH Terms] OR “tube*”[Text Word] OR “tubing”[Text Word]) AND “nephrostom*”[Text Word])) AND (“Postoperative Complications”[MeSH Terms] OR “post operat*”[Text Word] OR “infections”[MeSH Terms] OR “infect*”[Text Word] OR “adverse effects”[MeSH Subheading] OR “adverse effect*”[Text Word] OR “risk factors”[MeSH Terms] OR “risk*”[Text Word] OR “treatment outcome/adverse effects”[MeSH Terms] OR “injur*”[Text Word] OR “complicat*”[Text Word] OR “sepsis”[Text Word] OR “tube exchange”[Text Word] OR “tube replace*”[Text Word] OR “fail*”[Text Word] OR “failure”[Text Word])) OR (“nephrostomy, percutaneous/instrumentation”[MeSH Terms] AND (“catheters, indwelling/adverse effects”[MeSH Terms] OR “catheters/adverse effects”[MeSH Terms])) OR ((“Nephrotomy”[MeSH Terms] AND “Catheter-Related Infections”[MeSH Terms]) OR (“nephrotom*”[Text Word] AND (“tube size”[Text Word] OR “tube material”[Text Word] OR “catheter*”[Text Word]) AND (“infect*”[Text Word] OR “complicat*”[Text Word] OR “injur*”[Text Word] OR “risk*”[Text Word] OR “outcome*”[Text Word] OR “sepsis”[Text Word] OR “adverse effect*”[Text Word] OR “fail*”[Text Word] OR “failure”[Text Word])))) NOT “nephrolithotomy, percutaneous”[MeSH Major Topic]) AND “English”[Language]) NOT (“systematic review”[Publication Type] OR “meta analysis”[Publication Type] OR “systematic review*”[Title] OR “meta analys*”[Title] OR “literature review”[Title] OR “narrative review”[Title] OR “scoping review”[Title] OR “case report*”[Title] OR “case series”[Title] OR “case stud*”[Title]).

### Data collection

**Selection of studies**

All results identified in the search strategy will be imported into the software Covidence. Title and abstract review will be performed independently by two authors. Next, for all selected abstracts, the full text will be referenced and reviewed by two independent authors for inclusion or exclusion based on previously mentioned criteria. Should any disagreements occur throughout this process, a third author will be used to yield final decision. A PRISMA-P flow chart will depict the final set of inclusion and exclusion criteria. ^19^ Throughout the process, we will be collecting the study characteristics, outcomes, and any relevant effect modifiers.

**Study characteristics data**

Article title, corresponding author, publication date, study setting, all conflicts of interest, study design, patient demographics and comorbidities, and inclusion and exclusion criteria will be noted. All primary and secondary outcomes will be noted along with any levels of uncertainty.

### Bias risk assessments

Bias will be assessed independently for each study that will be included in our systematic review. Like previously mentioned methods, two reviewers will independently assess risk with a third reviewer to yield final decision as necessary. The Cochrane Risk of Bias tool will be utilized to evaluate randomized control trials. ^20^ Cohort studies will be assessed using the ROBINS-II tool across their specified domains. ^21^

### Data synthesis

**Summary of study characteristics**

We will use descriptive statistics to give an overview of the listed studies. Information on the study population’s characteristics, such as the different comparisons and design elements, will be included in this. Additionally, for each result, we will show a network diagram with the size of the nodes based on the patient count and the thickness of the edges based on the number of studies per comparison.

**Estimation of relative treatment effect**

We will compute the odds ratio for dichotomous outcomes, mean difference or normalized mean difference for continuous outcomes, and hazard ratios for time-to-event or survival outcomes to ascertain the relative treatment effects. The surface under the cumulative ranking curve or p-score, which offers a scalar value ranging from 0 (least effective) to 1 (most effective) for each therapy in respect to a particular outcome, will be used to rank the treatments overall for each outcome. ^22^

**Assessment of statistical inconsistency**

The back-calculation technique will be used to assess local inconsistencies. We will also evaluate global inconsistency using the design-by-treatment interaction model. ^23^ The R programming language and software will be used to carry out the consistency checks.

**Consideration of unit of analysis issues and missing data**

We will consider the correlation between effect estimates in the network and incorporate multi-arm studies. Even though we do not plan to include any cluster or crossover randomized controlled trials (RCTs), if any are, we will abide by the recommendations in the Cochrane Handbook for Systematic Review of Interventions. Every analysis will be done with the objective of treating each individual. For each included study, we will investigate the level of missing data and examine the approaches taken to deal with it in the risk of bias analysis. We will do a sensitivity analysis for the key outcomes to determine the effect of including studies with a significant quantity of missing data. Measures of uncertainty will, whenever possible, be converted into standard errors. If no certainty is provided or can be inferred, the mean of known standard errors from included studies will be calculated. ^24^

**Assessment of reporting biases**

To assess the possibility of reporting bias and small-study effects, we will visually inspect the funnel plots or each treatment where possible ( *i.e.*, at least 10 studies for a given treatment). Additionally, to evaluate small-study effects, we will assess comparison-adjusted funnel plots, and where possible, conduct meta-regression with study variance as the covariate. ^25^^,^^26^

When possible ( *i.e.*, when there are at least 10 trials for a given therapy), we will visually evaluate the funnel plots for each treatment to check for reporting bias and small-study effects. ^25^ Additionally, we will employ comparison-adjusted funnel plots (Chaimani *et al.*) and, if necessary, do meta-regression using study variance as the covariate to evaluate small-study effects. ^26^^–^^28^

### Exploring heterogeneity and inconsistency

For the primary outcomes, we will explore possible sources of heterogeneity, such as the indication for percutaneous nephrostomy, other comorbidities, and the method of outcome ascertainment. If enough studies are available, we will perform subgroup analyses based on these characteristics. Additionally, for the primary outcomes, we will conduct sensitivity analyses by:

Restricting the analysis to larger studies, as smaller studies may introduce bias. The study size will be determined by the median of the included studies, and in the sensitivity analysis, studies below the median size will be excluded.

Removing studies with more than 20% missing data.

### Descriptive synthesis & summary of findings

Descriptive synthesis and summary of findings will be performed in accordance with the Synthesis without Meta-Analysis in Systematic Reviews (SWiM) guidelines. ^29^ For each primary outcomes, two authors will independently assess the nine SWiM domains as very low, low, moderate, or high confidence. ^29^

## Conclusions

### Limitations

While this is a timely and important topic to explore as a systematic review, there are some limitations to this study. First, there potentially is only scant literature on the subject. As a result, conclusions drawn from the systematic review might not fully represent the entire clinical context of the role these interventions play on our outcome. Additionally, since this is a multicomponent analysis and only a descriptive synthesis will be performed through this systematic review, no relative effectiveness data will be collected. Additionally, it is possible that we will find statistically relevant results by chance alone; however, there are no methods to account for these issues in systematic reviews. Finally, different companies of these devices might manufacture their devices differently and with proprietary technology preventing a true one-to-one comparison across catheter materials and anti-dislodgement mechanisms. Given these limitations, we still strongly believe that the corresponding results will be meaningful to clinical practice but should be interpreted with caution.

### Summary

This systematic review will serve as the foundation for comparison of varying catheter tubes and sizes on the frequency of drainage catheter exchange interval. As the incidence of drainage procedures increases, there may be patients with certain comorbidities that may benefit from a shorter exchange interval.

### Ethics & dissemination

New institutional review board approval is not required for this study since it involves a systematic review of existing published data. Our intention is to disseminate our results through reputable peer-reviewed journals and conference presentations of significant influence. Given the growing prevalence of percutaneous drainage procedures, it is crucial for healthcare practitioners involved in the management of these patients to have access to concise and synthesized educational resources on this subject. The data synthesized from our study will be made accessible to readers.

### Patient and public involvement

This protocol was developed without the involvement of any patients or members of the public, and their inclusion in the completion or dissemination of the corresponding review and results is not planned.

### Study status

We plan to begin title/abstract review upon successful submission of this protocol.

## Data Availability

No data are associated with this article. Figshare: PRISMA-P checklist for ‘Impact of drainage catheter material, size, and anti-dislodgement mechanism on percutaneous nephrostomy exchange intervals: a systematic review protocol’, https://doi.org/10.6084/m9.figshare.23937171.v1. ^30^ Data are available under the terms of the Creative Commons Attribution 4.0 International license (CC-BY 4.0).
