# Supplementary material for: Impact of drainage catheter material, size, and anti-dislodgement mechanism on percutaneous nephrostomy exchange intervals: a systematic review protocol
Source: F1000Res. 2023 Oct 30;12:1417. [Version 1] doi: 10.12688/f1000research.135431.1 (PMC10905141; doi:10.12688/f1000research.135431.1)
Supplement: Peer review history

# Reviewer report: Reviewer response for version 1

**DOI:** 10.5256/f1000research.148548.r244991

**Published:** 2024-02-20

**Authors:** Dragos Puia

**Affiliations:** 1 University for Medicine and Pharmacy, Iasi, Romania

Dear Researchers, I think your research is of interest considering that there are many patients with PCN, and many have problems with the catheter. However, the patients' pathology is very heterogeneous, and the underlying disease could influence the outcome. I would suggest performing a subgroups' analysis according to their underlying disease (cancers, kidney stones, other disease) and also if they have received a specific treatment like chemotherapy (in these patients there is high catabolism with an increased excretion of uric acid)

Is the study design appropriate for the research question?

Yes

Is the rationale for, and objectives of, the study clearly described?

Yes

Are sufficient details of the methods provided to allow replication by others?

Yes

Are the datasets clearly presented in a useable and accessible format?

Yes

Reviewer Expertise:

Urology

I confirm that I have read this submission and believe that I have an appropriate level of expertise to confirm that it is of an acceptable scientific standard.

# Reviewer report: Reviewer response for version 1

**DOI:** 10.5256/f1000research.148548.r234532

**Published:** 2024-02-08

**Authors:** Azza Aly

**Affiliations:** 1 Medical surgical nursing, Associate professor in medical surgical nursing, Damanhour, Egypt

Dear Researchers 

 I appreciate your efforts in preparing this protocol However, I have some points that provide specific areas for improvement and clarification to enhance the scientific integrity of the systematic review. The protocol is well structured, but the extensive technical details would benefit from simplification for a broader understanding.

 regarding the Methodology Provide a clear rationale for the chosen methodologies, addressing potential biases and limitations.

 I think you need to engage the patients in the research process to enhance the relevance of the study and its applicability to real-world scenarios.

 Contain the current status of the study or any progress made since the protocol was submitted.

Is the study design appropriate for the research question?

Yes

Is the rationale for, and objectives of, the study clearly described?

Yes

Are sufficient details of the methods provided to allow replication by others?

Yes

Are the datasets clearly presented in a useable and accessible format?

Yes

Reviewer Expertise:

medical surgical nursing

I confirm that I have read this submission and believe that I have an appropriate level of expertise to confirm that it is of an acceptable scientific standard.
